# Impact of early postoperative blood glucose variability on serum endocan level in cardiac surgery patients: a sub study of the ENDOLUNG observational study

**DOI:** 10.1186/s12933-023-01959-5

**Published:** 2023-08-24

**Authors:** Etienne Chazal, Lucas Morin, Sidney Chocron, Philippe Lassalle, Sebastien Pili-Floury, Lucie Salomon du Mont, David Ferreira, Emmanuel Samain, Andrea Perrotti, Guillaume Besch

**Affiliations:** 1 Université de Franche-Comté, CHU Besançon, EA 3920, Département d’Anesthésie Réanimation Chirurgicale, Besançon, F-25000 France; 2 CHU Besançon, Inserm CIC 1431, Besançon, F-25000 France; 3 Université de Franche-Comté, CHU Besançon, EA 3920, Service de Chirurgie Cardiaque, Besançon, F-25000 France; 4 Univ. Lille, CNRS, Inserm, CHU Lille, Institut Pasteur de Lille, U1019-UMR9017-CIIL-Centre d’Infection et d’Immunité de Lille, Équipe immunité pulmonaire, Biothelis, Lille, F-59000 France; 5 Université de Franche-Comté, CHU Besançon, EA 3920, Service de Chirurgie Vasculaire et Endovasculaire, Besançon, F-25000 France; 6 Université de Franche-Comté, CHU Besançon, EA 481 Neuroscience, Département d’Anesthésie Réanimation Chirurgicale, Besançon, F-25000 France

**Keywords:** Glycemic variability, Cardiac surgery, Endothelial function, Endocan

## Abstract

**Background:**

Early postoperative glycemic variability is associated with worse outcome after cardiac surgery, but the underlying mechanisms remain unknown. This study aimed to describe the relationship between postoperative glycemic variability and endothelial function, as assessed by serum endocan level in cardiac surgery patients.

**Methods:**

We performed a *post hoc* analysis of patients included in the single-center observational ENDOLUNG study. Adult patients who underwent planned isolated coronary artery bypass graft surgery were eligible. Postoperative glycemic variability was assessed by calculating the coefficient of variability (CV) of blood glucose measured within 24 (CV_24_) and 48 (CV_48_) hours after surgery. Serum endocan level was measured at 24 (Endocan_24_) and 48 (Endocan_48_) hours after surgery. Pearson’s correlation coefficient with 95% confidence interval (95% CI) was calculated between CV_24_ and Endocan_24_, and between CV_48_ and Endocan_48_.

**Results:**

Data from 177 patients were analyzed. Median CV_24_ and CV_48_ were 18% (range 7 to 39%) and 20% (range 7 to 35%) respectively. Neither CV_48_ nor CV_24_ were significantly correlated to Endocan_48_ and Endocan_24_ respectively (r (95% CI) = 0.150 (0.001 to 0.290; and r (95% CI) = 0.080 (-0.070 to 0.220), respectively).

**Conclusions:**

Early postoperative glycemic variability within 48 h after planned cardiac surgery does not appear to be correlated with postoperative serum endocan level.

**Clinical trial registration number:**

NCT02542423.

## Background

Early postoperative glycemic variability, defined as the fluctuation of blood glucose values within or outside of a target range, has been shown to be associated with impaired outcomes after cardiac surgery and transcatheter aortic valve implantation (TAVI), as well as in patients hospitalized for acute heart failure, regardless of the quality of blood glucose control obtained during the same period [1–3]. Glycemic variability was reported to independently increase the rate of postoperative complications, the length of stay and the mortality in the intensive care unit (ICU) and mortality in patients undergoing coronary artery bypass grafting (CABG) [3, 4]. The underlying mechanisms that could explain the deleterious effect of glycemic variability during the perioperative period have never been specifically investigated. Previous studies suggested that glycemic variability could increase oxidative stress and endothelial dysfunction in non-acutely ill patients [5–7], but no study has addressed this issue after cardiac surgery. Yet, postoperative glycemic variability is reportedly mainly associated with major adverse cardiovascular events after TAVI or CABG, suggesting that early postoperative glycemic variability could impair the endothelial function in this population [2, 3].

Endocan (or Endothelial-cell Specific Molecule-1 (ESM-1) is a proteoglycan that is produced and secreted by endothelial cells [8]. Since it mainly arises from lungs, serum endocan levels were initially shown to identify early acute lung injury in critically ill patients [9]. Results from the ENDOLUNG study suggested that serum endocan level could be an early marker of postoperative pneumonia after cardiac surgery [10]. More recently, serum endocan level was associated with endothelial dysfunction in atherosclerosis and in diabetic patients, and with major adverse cardiovascular events after acute coronary syndrome [8, 11–16]. The kinetic of serum endocan level after cardiac surgery appeared to be related to the vasoplegic syndrome that could be considered as the clinical expression of postoperative endothelial dysfunction [17]. Moreover, brachial artery flow-mediated dilatation, which is a reference method to non-invasively measure endothelial dysfunction, was described to be correlated to serum endocan level, suggesting that serum endocan level could be a reliable biomarker for endothelial dysfunction [18]. We therefore hypothesized that: [1] early postoperative glycemic variability could alter endothelial function after cardiac surgery, and [2] serum endocan level could be a good proxy measure for endothelial dysfunction after cardiac surgery.

This purpose of this study was thus to investigate whether early post-operative glycemic variability was positively associated with serum endocan levels after cardiac surgery.

## Methods

### Data source and study design

The prospective Endocan Predictive Value in Postcardiac Surgery Acute Respiratory Failure (ENDOLUNG) study was a single-center observational cohort study, which prospectively enrolled adult patients undergoing elective cardiac surgery between January 1 and May 23, 2016. Patients who met any of the following criteria were excluded: pregnant and breastfeeding women, patients who were unable to give informed consent, who had emergency surgery, or who had on-going pulmonary infection, inflammation, or an active malignancy. The rationale and methods have previously been described elsewhere [10]. Briefly, the primary aim of the ENDOLUNG study was to evaluate whether endocan could be a useful and accurate marker to predict acute respiratory failure after cardiac surgery. Patients were included at the time of hospital admission and were then followed up during surgery and throughout their postoperative stay (range 6–83 days). All-cause mortality after hospital discharge was determined through deterministic record linkage with the National Registry of Deceased Persons.

### Study population

This sub-analysis included all patients enrolled in the ENDOLUNG study who had complete data regarding glycemia (fasting blood glucose) at baseline and during the first 48 h after surgery.

### Measurement of postoperative blood glucose levels and glycemic variability

After their admission into the cardiac surgical intensive care unit, patients received intravenous glucose infusion administered at a constant rate of 4.0 to 4.5 g per hour until oral feeding was resumed. Blood glucose control was performed using a validated, nurse-led intravenous insulin therapy protocol, whereby the infusion rate was adjusted dynamically to achieve a blood glucose target of 100–139 mg.dl^− 1^ (5.5–7.7 mmol.l^− 1^). All blood glucose values were obtained from glucose meter reading (Optium Xceed™, Abbott Diabetes Care Ltd., Witney, UK) measured from arterial blood samples drawn from the indwelling arterial catheter. Blood glucose levels were checked every hour until 12 consecutive blood glucose values fell within the target range, then every 3 h. Hourly blood glucose measurement was resumed if there was a change in the insulin infusion rate, in the patient’s clinical condition, or if vasopressors or renal replacement therapy were either initiated or discontinued. Intravenous insulin infusion was replaced by subcutaneous insulin as soon as oral feeding was reinstated, and the medical team was consulted in case of severe hypoglycemia or uncontrolled hyperglycemia.

In the present study, our main exposure was postoperative glycemic variability during the 24 and 48 h after cardiac surgery. Although several methods have been proposed to describe glycemic variability, the coefficient of variation (CV) of blood glucose levels is now widely considered as the metric of choice to describe intraday fluctuation in glycemic variability. The CV was calculated by dividing the standard deviation of all blood glucose level measurements during the first 24 and 48 h post-operatively by the mean blood glucose level during the same time window, expressed as percentage. Glycemic variability is hereafter expressed as a percentage. Because the ENDOLUNG study protocol only required blood glucose levels at 6, 24, 48 and 72 h after surgery, we enriched the initial dataset by retrospectively reviewing original patient charts and extracting each hourly blood glucose measurement for the first 48 h of follow up. This allowed us to refine the time resolution of our estimates for intra-day glycemic variability.

### Outcomes assessment

Endocan blood levels at 24 and 48 h after cardiac surgery were considered as our co-primary outcomes. Blood samples for serum endocan level measurements were collected in 5 ml-EDTdA tubes and centrifuged at 3000 rpm for 10 min at room temperature (18–25 °C). They were aliquoted in 0.5mL tubes (Eppendorf, Le Pecq, France) and frozen at -20 °C until assayed. Serum endocan level was measured using the Lunginnov ELISA kit (EndoMark® H1) based on immunoenzymatic assay (Lunginnov SAS, Lille, France) using a quality control sample targeted at 3.5 ng/mL, between-assay imprecision was estimated to be ~ 12%.

### Measurement of individual patient characteristics

Sex, age, body mass index (BMI), comorbidities (e.g.·medical history of diabetes, hypertension, dyslipidemia, peripheral arterial disease, carotid stenosis, chronic respiratory disease or cardiac arrythmia), smoking status, left ventricular ejection fraction (LVEF), EuroScore, estimated glomerular filtration rate (eGFR), preoperative fasting blood glucose and glycosylated hemoglobin (HbA1c) at baseline were collected prospectively from the patients’ medical records. Perioperative data related to the surgical procedure (e.g. duration of extracorporeal circulation, duration of aortic clamping, blood loss and blood transfusion) were extracted from the computerized anesthesia monitoring software. Treatments administered in the intensive care unit and postoperative in-hospital complications (blood transfusion, atrial fibrillation, stroke, acute kidney injury, dialysis, pneumonia, or death) were also documented.

### Statistical methods

We first describe intra-day glycemic variability during the first 24 h post-surgery by plotting the minimum, maximum and mean blood glucose values measured in each individual patient. Since cut-off values sometimes suggested to define high variability (typically, CV ≥ 36%) were deemed unsuitable in the setting of postoperative intensive care, where glycemia is tightly monitored and controlled, we categorized patients into tertiles of the CV.

Second, to elucidate the hypothesized relationship between glycemic variability and endocan blood levels 24 and 48 h after surgery, we represented individual values for diabetic and non-diabetic patients on a scatterplot and fitted a two-way fractional-polynomial prediction curve. We also computed Pearson’s correlation coefficient with 95% confidence interval (CI) using Fisher’s z transformation. Finally, we conducted a non-prespecified subgroup analysis to explore a potential source of heterogeneity in the relationship between glycemic variability and endocan blood level. To this end, the correlation coefficient was stratified according to diabetes status, levels of HbA1c, BMI, eGFR, the existence of postoperative complications, the duration of extracorporeal circulation, and the EuroScore at baseline.

### Ethical approval

The ENDOLUNG study was approved by the Institutional Review Board (Comité de Protection des Personnes (CPP) Est II, under the number 10/544) and was registered on ClinicalTrials.gov (NCT02542423). All patients provided written informed consent.

## Results

Of the 186 patients enrolled in the ENDOLUNG study, 177 were included in the present study (9 patients had incomplete data regarding glycemia). Baseline characteristics and in-hospital postoperative outcomes are presented in Table 1. Age at enrollment was 69 (SD 9) years. Seventy (39%) patients had peripheral artery disease, 59 (33%) were diabetic, 134 (76%) were treated for hypertension and 148 (84%) underwent on-pump surgery. On average, patients had 37.2 (SD 4.5) blood glucose measurements during the first 48 h after surgery, with a mean blood glucose value of 135 (SD 12) mg.dl^− 1^ (7.4 (0.7) mmol.l^− 1^).

**Table 1:** Baseline characteristics of the study population

	n = 177 patients
Age, years (mean (SD))	69 (9)
N (%)	
< 55 years	11 (6)
55–64 years	40 (23)
65–74 years	74 (42)
75–84 years	44 (25)
≥ 85 years	8 (4)
Sex, N (%)	
Male	134 (76)
Female	43 (24)
EuroSCORE II, mean (SD)	5 (2)
Body mass index, N (%)	
< 25 kg.m^− 2^	57 (32)
25–30 kg.m^− 2^	71 (40)
> 30 kg.m^− 2^	49 (28)
Chronic comorbidities, N (%)	
Hypertension	134 (76)
Dyslipidemia	99 (56)
Diabetes mellitus	59 (33)
Atrial fibrillation	30 (17)
Peripheral artery disease	70 (39)
Current smokers, N (%)	100 (57)
Fasting blood glucose in mg/dl, mean (SD)	119 (40)
Glycated hemoglobin (HbA1c), % (mean (SD))	6.4 (1.2)
Estimated creatinine clearance, N (%)	
< 60 mL/min/1.73 m^2^	35 (20)
60–89 mL/min/1.73 m^2^	89 (50)
≥ 90 mL/min/1.73 m^2^	53 (30)
Left ventricular ejection fraction, N (%)	
≤ 30%	6 (4)
31–50%	42 (25)
> 50%	119 (71)
On-pump surgery, N (%)	148 (84)
Duration of extracorporeal circulation, No (%)	
< 60 min	30 (20)
60–89 min	63 (43)
≥ 90 min	55 (37)
Duration of aortic cross-clamping, in minutes (mean (SD))	72 (32)
Postoperative complications, N (%)	
Any complication	85 (48)
Blood transfusion	81 (46)
Atrial fibrillation	73 (41)
Stroke	1 (1)
Acute kidney injury	24 (14)
Dialysis	4 (2)
Pneumonia	16 (9)
ICU length of stay in days (mean (SD))	5 (4)
In-hospital length of stay in days (mean (SD))	12 (8)
In-hospital death, N (%)	3 (2)

SD: standard deviation; N: number of patients; ICU: intensive care unit

**Fig. 1:**
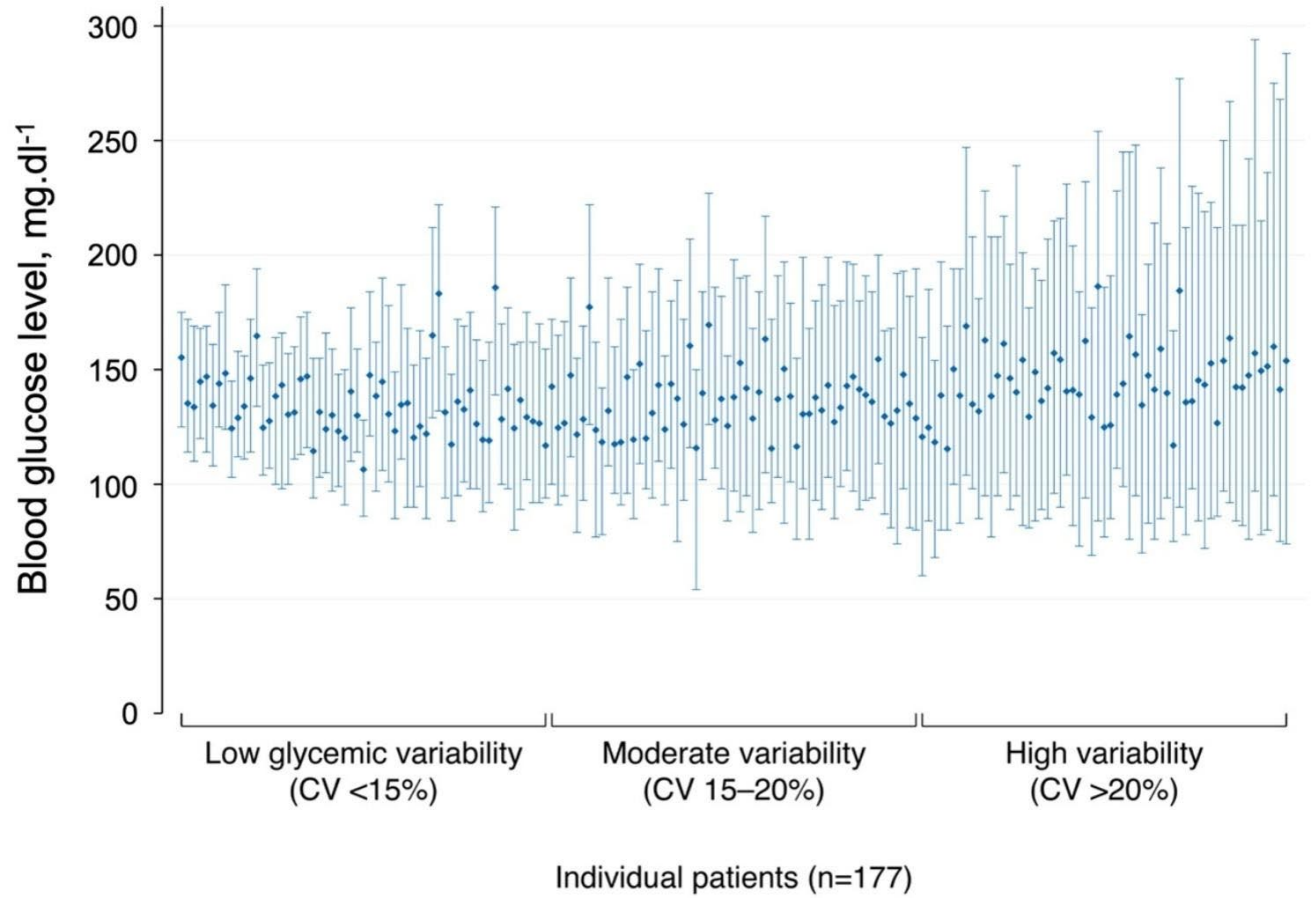
Glycemia and coefficient of variability of blood glucose within 48 h after surgery. CV is the coefficient of variability of blood glucose, defined as the ratio of the standard deviation of blood glucose to the average of blood glucose expressed in percent. Each bar represents the minimum, maximum and mean blood glucose values measured within 48 h after surgery in each patient included in the study

Figure 1 shows the minimum, maximum and mean blood glucose value measured in each individual patient, plotted in ascending order of the CV of blood glucose. The median coefficient of glycemic variability during the first 24 and 48 h after surgery was respectively 18% (range 7 to 39%) and 20% (range 7 to 35%).

**Fig. 2:**
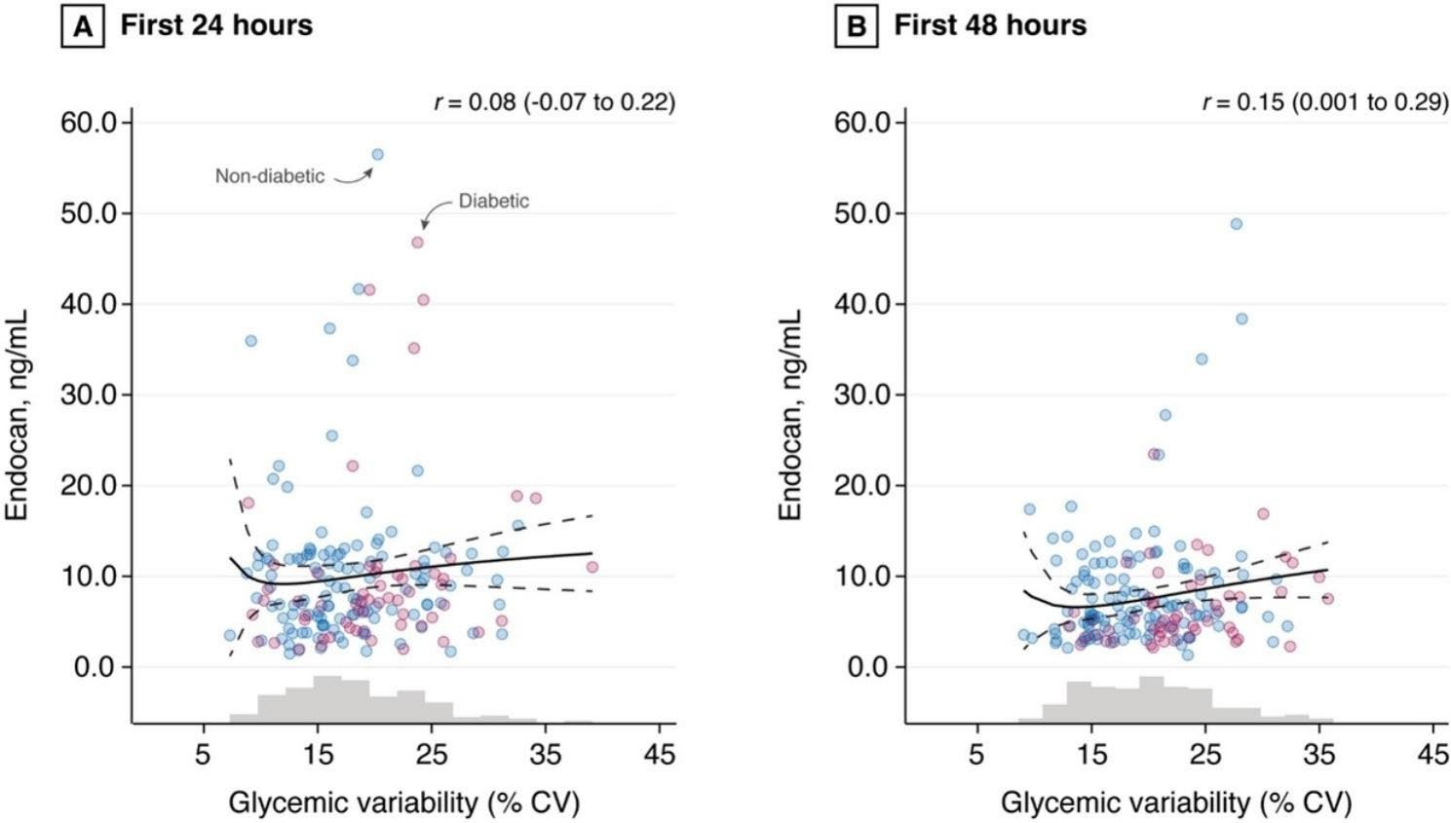
Scatter plots describing coefficient of variability of blood glucose and serum endocan level within (**A**) 24 and (**B**) 48 h after surgery. Each point represents an individual patient. Blue points figure non-diabetic patients and red points patients with a known medical history of diabetes mellitus. Gray histograms represent the distribution of glycemic variability within the entire cohort

**Fig. 3:**
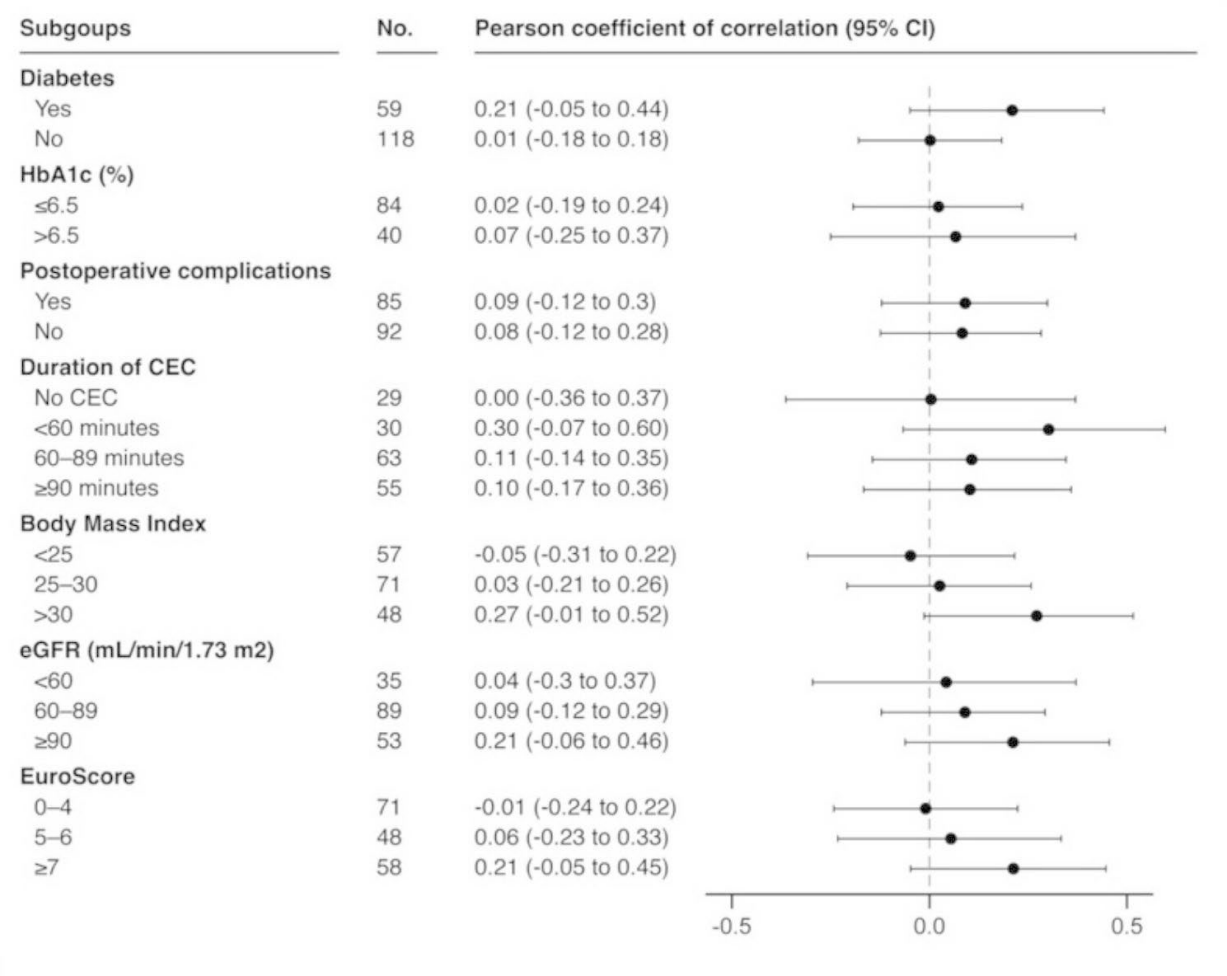
Forest-plot showing the correlation between the coefficient of variability of blood glucose and serum endocan level at 48 h after surgery in the non-prespecified subgroups

The scatter plots showing the CV of blood glucose and serum endocan levels within 24 and 48 h after surgery are presented in Fig. 2A and B respectively. Neither the CV within 24 h nor the CV within 48 h after surgery was significantly correlated with serum endocan level at 24 and 48 h after surgery respectively (r = 0.08, 95% CI -0.07 to 0.22, and r = 0.15, 95% CI 0.001 to 0.290, respectively) (Fig. 2A and B). No clinically meaningful difference was observed between diabetic and non-diabetic patients, or any other subgroup analyzed, including on-pump versus off-pump surgery (Fig. 3).

## Discussion

The results of the present study suggest that early postoperative glycemic variability was not associated with postoperative serum endocan level, neither after on-pump nor after off-pump cardiac surgery, either in non-diabetic or in diabetic patients.

Previous studies suggested that glycemic variability could promote endothelial dysfunction, as measured using brachial artery flow-mediated dilation assessed with doppler ultrasonography in non-acutely ill diabetic and non-diabetic patients [7, 19, 20]. Increased serum endocan level was reported to be related to endothelial dysfunction in diabetic patients [8, 13–16]. Serum endocan level appeared to be significantly correlated to brachial artery flow-mediated dilation [18], which is a validated and widely-used non-invasive method of measuring endothelial function [12, 21, 22]. Our study is the first to investigate the relationship between early postoperative glycemic variability and postoperative endothelial dysfunction assessed by serum endocan level in patients undergoing cardiac surgery. Several reasons can be put forward to explain the lack of significant correlation between early postoperative glycemic variability and serum endocan level observed in the present study.

First, the glycemic variability observed in the present cohort was quite low compared to the level reported in studies describing the association between glycemic variability and impaired prognosis in cardiac surgery patients, and between glycemic variability and endothelial dysfunction in non-acutely ill patients [3, 5]. This low postoperative glycemic variability could result from the high quality of blood glucose control provided by the insulin therapy protocol used in the cardiac surgery intensive care unit [23, 24]. Intravenous insulin therapy is recommended to avoid blood glucose level above 150 mg.dl^− 1^ (8.2 mmol.l^− 1^) and below 100 mg.dl^− 1^ (5.5 mmol.l^− 1^) after cardiac surgery since intra and postoperative hyperglycemia was reported to increase postoperative morbidity and mortality [25, 26]. Lowering blood glucose levels during the postoperative period could have contributed to decreased serum endocan levels [27].

Another explanation could be that the postoperative glycemic variability observed in the present study did not reach a sufficient threshold to promote postoperative endothelial dysfunction. However, no correlation between the CV of blood glucose and serum endocan level was observed even in patients presenting the highest postoperative glycemic variability.

Third, serum endocan might not be the most appropriate biomarker to describe the endothelial dysfunction related to glycemic variability. Serum endocan was initially described to be related to pulmonary endothelial dysfunction and no previous study reported that glycemic variability could specifically alter the pulmonary circulation. A previous study suggested that serum endocan level could be useful for early diagnosis of subclinical atherosclerosis in type 2 diabetes mellitus [28]. Moreover, serum endocan level was reported to be overexpressed in patients with stress hyperglycemia having acute ST-segment elevation myocardial infarction (STEMI) [29] and to be higher in diabetic patients with acute STEMI compared to newly diagnosed, untreated and uncomplicated type 2 diabetes mellitus patients [30]. However, endothelial dysfunction related to glycemic variability in patients with diabetes is a complex disorder involving several underlying mechanisms that could be imperfectly mirrored by serum endocan levels [31]. Different results might be observed if other biomarkers were used to investigate postoperative endothelial dysfunction or oxidative stress.

Fourth, the synthesis and secretion of endocan is upregulated by pro-inflammatory cytokines such as interleukin-6 (IL-6), IL-1β or tumor necrosis factor-α (TNF-α) and downregulated by anti-inflammatory factors, such as IL-10 or interferon-γ [8]. On-pump and off-pump cardiac surgery is responsible for an intense systemic inflammatory reaction, including the release of high levels of pro-inflammatory cytokines, such as IL-6, IL-1β and TNF-α. This in turn could stimulate the secretion of endocan without any further trigger [32]. Furthermore, the postoperative spontaneous fluctuations of blood glucose levels were actively modified using insulin therapy protocol to avoid potentially deleterious hyperglycemia. Thus, the relationship between serum endocan level and early postoperative glycemic variability observed in the present study could be biased by postoperative systemic inflammation and blood glucose control.

Finally, we cannot rule out the possibility that the underlying mechanisms explaining the deleterious impact of early postoperative glycemic variability in cardiac surgery patients could differ from the mechanisms described in non-acutely ill diabetic patients.

The present study has some limitations. First, postoperative glycemic variability was not measured using a continuous monitoring system and may have been underestimated [33]. However, blood glucose was closely monitored in the 48 h after surgery using an insulin therapy protocol used in the cardiac surgery intensive care unit, and nearly one blood glucose value per hour was obtained for each patient during the study period. Second, the timing of measurement of serum endocan level may have been inappropriate to accurately measure the impact of postoperative glycemic variability on endothelial function, since the ENDOLUNG study was not designed to investigate this hypothesis. However, no difference was observed in the different subgroup analyses, and we can assume that no correlation exists between early postoperative glycemic variability and serum endocan level after planned cardiac surgery. Third, the infusion of catecholamines was associated with both increased serum endocan level and increased blood glucose level [17]. Thus, the infusion of catecholamines could have impaired the potential relationship between serum endocan level and glycemic variability. Unfortunately, neither the dose nor the duration of catecholamines infusion were collected in the present study.

## Conclusions

Early postoperative glycemic variability does not appear to be correlated with postoperative serum endocan level at 48 h after planned cardiac surgery. Several conditions that are commonly observed after cardiac surgery could have affected the kinetic of serum endocan level and could explain that the primary hypothesis of the study was not confirmed. Furthermore, endocan might not be the most appropriate biomarker to investigate endothelial dysfunction during the postoperative period in patients undergoing elective cardiac surgery. Further studies are warranted to identify the underlying mechanisms that contribute to the deleterious impact of postoperative glycemic variability, which could represent a potential new therapeutic target to improve the prognosis of cardiac surgery patients.

## Data Availability

Data and documents used and/or analyzed during the current study will be made available on reasonable request after approval by an Institutional Committee of the aim and methodological plan proposed by the researchers. Proposal should be directed to the Direction à la Recherche Clinique et à l'Innovation, CHU Besançon, France (e-mail address: dam@chu-besancon.fr).

## Abbreviations

TAVI: transcatheter aortic valve implantation
ICU: intensive care unit
ESM-1: Endothelial-cell Specific Molecule-1
CV: coefficient of variation
BMI: Body Mass Index
LVEF: Left Ventricular Ejection Fraction
eGFR: estimated Glomerular Filtration Rate
HbA1c: glycosylated hemoglobin
CI: Confidence Interval
